# ATP Alters the Oxylipin Profiles in Astrocytes: Modulation by High Glucose and Metformin

**DOI:** 10.3390/brainsci15030293

**Published:** 2025-03-11

**Authors:** Alexey I. Drozhdev, Vladislav O. Gorbatenko, Sergey V. Goriainov, Dmitry V. Chistyakov, Marina G. Sergeeva

**Affiliations:** 1 Faculty of Bioengineering and Bioinformatics, Lomonosov Moscow State University, 119234 Moscow, Russia; dr.alexey7@gmail.com (A.I.D.); vansquirrel24@gmail.com (V.O.G.); 2 Institute of Pharmacy and Biotechnology, Peoples’ Friendship University of Russia (RUDN University), 117198 Moscow, Russia; goryainov-sv@rudn.ru; 3 Belozersky Institute of Physico-Chemical Biology, Lomonosov Moscow State University, 119992 Moscow, Russia; mg.sergeeva@gmail.com

**Keywords:** ATP, astrocytes, oxylipins, hyperglycemia, inflammation, metformin

## Abstract

**Background:** Astrocytes play a key role in the inflammatory process accompanying various neurological diseases. Extracellular ATP accompanies inflammatory processes in the brain, but its effect on lipid mediators (oxylipins) in astrocytes remains elusive. Metformin is a hypoglycemic drug with an anti-inflammatory effect that has been actively investigated in the context of therapy for neuroinflammation, but its mechanisms of action are not fully elucidated. Therefore, we aimed to characterize the effects of ATP on inflammatory markers and oxylipin profiles; determine the dependence of these effects on the adaptation of astrocytes to high glucose levels; and evaluate the possibility of modulating ATP effects using metformin. **Methods:** We estimated the ATP-mediated response of primary rat astrocytes cultured at normal (NG, 5 mM) and high (HG, 22.5 mM) glucose concentrations for 48 h before stimulation. Cell responses were assessed by monitoring changes in the expression of inflammatory markers (TNFα, IL-6, IL-10, IL-1β, iNOS, and COX-2) and the synthesis of oxylipins (41 compounds), assayed with ultra-high-performance liquid chromatography and tandem mass spectrometry (UPLC-MS/MS). Intracellular pathways were assessed by analyzing the phosphorylation of p38; ERK MAPK; transcription factors STAT3 and NF-κB; and the enzymes mediating oxylipin synthesis, COX-1 and cPLA2. **Results:** The stimulation of cells with ATP does not affect the expression of pro-inflammatory markers, increases the activities of p38 and ERK MAPKs, and activates oxylipin synthesis, shifting the profiles toward an increase in anti-inflammatory compounds (PGD2, PGA2, 12-HHT, and 18-HEPE). The ATP effects are reduced in HG astrocytes. Metformin potentiated ATP-induced oxylipin synthesis (11-HETE, PGD2, 12-HHT, 15-HETE, 13-HDoHE, and 15-HETrE), which was predominantly evident in NG cells. **Conclusions:** Our data provide new evidence showing that ATP induces the release of anti-inflammatory oxylipins, and metformin enhances these effects. These results should be considered in the development of anti-inflammatory therapeutic approaches aimed at modulating astrocyte function in various pathologies.

## 1. Introduction

ATP is not only the main source of energy in the mammalian body but also enables the transmission of signals between cells. Nearly all living organisms, from bacteria to mammals, are capable of releasing ATP into the intercellular space for signal transduction, and many cells possess receptors for extracellular ATP called P2-purine receptors [1,2,3,4]. Purinergic signaling cascade and ATP release processes play a special role in astrocytes [1,5,6]. In many pathologic processes in the central nervous system (CNS), there is a rapid release of large amounts of ATP from astrocytes in the pericellular space, which can reach μM concentrations [7]. Astrocytes are actively involved in intercellular communication [5,8]. For this, astrocytes release chemical messengers, called gliotransmitters [9,10]. Gliotransmitters are increasingly studied for their role in neuroinflammation and neurodegenerative diseases [8]. ATP plays an important role as a gliotransmitter [9,10,11], but the mechanisms of ATP’s participation as a signaling substance in the inflammatory processes of astrocytes are not well understood.

Astrocytes play essential roles in nervous system homeostasis and are the fulcrum of inflammatory complications accompanied by various neurological diseases [12]. Upon the activation of toll-like receptors (TLR), astrocytes produce various substances, including proteins (both pro- and anti-inflammatory cytokines) and oxylipins, derivatives of polyunsaturated fatty acids (PUFAs) [12,13,14]. Although ATP synthesis, its autocrine effects via purinergic receptors on astrocytes, and the effects of this process on neurons have been studied [11,15,16], there are no data on the effects of extracellular ATP as a stimulus for the synthesis of various oxylipins. Oxylipins are signaling lipids produced through the action of multiple enzymatic and non-enzymatic pathways, including cyclooxygenase (COX), lipoxygenase (LOX), and epoxygenase (CYP). These PUFAs are converted simultaneously, resulting in a mixture of active compounds [17,18]. These compounds can both enhance the inflammatory response (pro-inflammatory markers) and attenuate it (anti-inflammatory compounds), and some of these compounds have been linked to the resolution process [17,19]. This leads to the fact that it is not possible to assess the specificity of the response by analyzing the changes in an individual compound. The development of modern analytical methods has advanced to a degree that allows for the simultaneous detection and quantification of oxylipins (oxylipin profiles) [20]. It has been shown that oxylipin profiles in astrocytes can be both pro- and anti-inflammatory, depending on the stimulus [21,22,23]. The characterization of the effect of extracellular ATP on oxylipin profiles will allow us to assess its involvement in the inflammatory response of astrocytes.

It is well known that hyperglycemia alters the innate immune system of an organism, including brain function and pathology [24,25]. Astrocytes are the key to glucose metabolism in the brain [25]. It has been shown that high glucose concentrations can have a significant impact on astrocyte metabolism [15,26,27], shifting the cellular response toward the increased expression and release of pro-inflammatory cytokines [26,27] and pro-inflammatory oxylipins [21]. At the same time, the processes of influence of glucose concentration on the purinergic signaling cascade of astrocytes remain poorly understood.

Understanding the relationship between energy metabolism and responses to pro-inflammatory stimuli provides a basis for exploring the potential use of hypoglycemic drugs as anti-inflammatory agents [28]. Metformin is the first-line treatment for type 2 diabetes, with a beneficial effect on neuroinflammatory pathologies [28]. In vivo studies using various models of neurological diseases have demonstrated the significant role of astrocytes in the effects of metformin [29]. These findings have prompted further in vitro investigations into how metformin influences various functions of astrocytes [30,31,32]. The cellular effects of metformin indicate its anti-inflammatory properties, including a decrease in pro-inflammatory marker expression induced by TLR stimuli [33,34]. In light of this, it would be interesting to evaluate the effect of metformin on pro-inflammatory markers and oxylipins when using extracellular ATP as a stimulus.

Thus, the aims of this work are (1) to characterize the effects of ATP on inflammatory markers and oxylipin profiles; (2) to determine the dependence of ATP effects on cellular adaptation to high glucose; and (3) to evaluate the possibility of interaction between metformin and ATP effects.

## 2. Materials and Methods

### 2.1. Primary Cell Culture

Cells were obtained from two-day-old pups of Wistar rats (both sexes). In brief, the brains from decapitated pups were triturated against nylon meshes with pores of 250 and 136 μm. The cells were cultured in DMEM (#21885-025, Gibco, Thermo Fisher Scientific, Waltham, MA, USA), 1 g/L D-glucose, 10% fetal bovine serum (FBS, One Shot, Gibco, ref. A3160801, lot 2166297), 50 units/mL streptomycin, and 50 μg/mL penicillin at 37 °C, with 10% CO_2_. After five days of cultivation, the culture medium was replaced with a fresh medium and shaken at 200 rpm for 4 h to remove microglia. The astrocyte-enriched cultures were further grown for the following four days, and the medium was replaced every 2 days and maintained for 2 days in DMEM with a 5 mM glucose (#21885-025, Gibco, NG) or 22.5 mM glucose (#41966-029, Gibco, HG) concentration. In these cultures, more than 95% of the cells were positive for the glial fibrillary acidic protein astrocyte marker, and <2% were positive for isolectin B4, a microglia-specific marker [35]. Cells were then stimulated with metformin (2.5 mM, hydrochloride, #13118, Cayman Chemical, Ann Arbor, MI, USA) or ATP (100 μM, #1191, Sigma-Aldrich, St. Louis, MO, USA).

### 2.2. Measurement of the Relative RNA Expression Level

Total mRNA was extracted using the GeneJET RNA Purification Kit (Thermo Scientific, Waltham, MA, USA). The concentration of RNA was measured using an Implen NanoPhotometer C (Westlake Village, CA, USA). cDNA was generated according to the manufacturer’s instructions using the MMLV RT kit (Evrogen, Moscow, Russia) with oligo-(dT)-primers. qPCR was performed using the 5 × PCR-HS-SYBR mix (Evrogen, Moscow, Russia) and the DTlite 4 thermocycler (DNATechnology, Moscow, Russia). The results were normalized at the β-actin mRNA level. The primer sequences were synthesized as shown in Table 1.

### 2.3. Western Blot Analysis

Western blot analysis was performed according to a protocol, as described previously [21]. In brief, astrocytes were lysed with modified radioimmunoprecipitation assay (RIPA) buffer (50 mM Tris, 1% NP-40, 0.25% Na-deoxycholate, 150 mM NaCl, 1 mM EDTA, 1 mM Na3VO4, 1 mM NaF, and pH 7.4) and a protease/phosphatase inhibitor cocktail (Roche Molecular Biochemicals, Mannheim, Germany). Each lane of a 10% SDS-polyacrylamide gel was loaded with 20 μg of protein diluted in standard Laemmli buffer, and electrophoresis was performed using conventional SDS-PAGE protocols. The protein concentration was determined by the Bradford assay. Antibodies against COX-1 (#4841), cPLA2 (# 2832), phospho-cPLA2 (Ser505) (#2831), p38 MAPK (#9212), phospho-p38 MAPK (Thr180/Tyr182), (#9211), p44/42 MAPK (Erk1/2, #9102), phospho-p44/42 MAPK Thr202/Tyr204 (Erk1/2, #9106), phospho-Stat3 (Tyr705) (D3A7) (#9145), STAT3 (#12640), NF-κB p65 (#8242), phospho-NF-κB p65 Ser536 (#3033), β-Tubulin, and secondary antibodies were from Cell Signaling Technology (Danvers, MA, USA). Following the blocking step, the membranes were incubated overnight at 4 °C in 0.05% Tween 20-phosphate-buffered saline (PBST) containing the appropriate primary antibody diluted at a ratio of 1:1000, with continuous agitation. Secondary horseradish-peroxidase-conjugated antibodies (1:10,000) were applied and incubated for 1 h at room temperature. The blots were visualized using the Pierce ECL Plus Western Blotting Substrate (Thermo Scientific, cat.no 32209, Waltham, MA, USA) and a ChemiDoc™ XRS+ gel documentation system (Bio-Rad, Hercules, CA, USA). Ratios of target antibody to β-tubulin were calculated for each sample.

### 2.4. UPLC-MS/MS Conditions and Sample Preparation

After the cell treatments, supernatants were collected and stored at −80 °C. The cell-free culture media were taken for solid-phase lipid extraction (Oasis^®^PRIME HLB cartridge (#186008056, Waters, Eschborn, Germany)). Oxylipins were analyzed using an ultra-performance liquid chromatography–tandem mass spectrometer (LCMS-8040 Triple quadrupole LC/MS/MS Shimadzu, Kyoto, Japan) in multiple-reaction monitoring mode at a unit mass resolution for both the precursor and product ions, as described previously [33]. The oxylipins standards were 6-keto PGF1α-d4 (#315210), TXB2-d4 (#319030), PGF2α-d4 (#316010), PGE2-d4 (#314010), PGD2-d4 (#312010), 5(S)-HETE-d8 (#334230), 12(S)-HETE-d8 (#334570), 15(S)-HETE-d8 (#334720), EPA-d5 (#10005056), DHA-d5 (#10005057), and ARA-d8 (#390010), from Cayman Chemical, Ann Arbor, MI, USA. For oxylipin analysis, lipid mediators were identified using the Lipid Mediator Version 2 software (Shimadzu, Japan) according to the manufacturer’s instructions, based on accurate mass, tandem mass spectrometric (MS/MS) behavior, and retention time.

### 2.5. Experimental Data Analysis and Statistics

The results are presented as mean ± standard deviation (SD). The normality of the data was assessed using the Shapiro–Wilk test. Pairwise comparisons between the two groups were analyzed using Student’s *t*-test. For comparisons across multiple groups, a two-way ANOVA with an interaction term was performed, followed by Tukey’s post hoc test for multiple comparisons. *p* < 0.05 was considered statistically significant. Three biological replicates were performed in all experiments.

## 3. Results

### 3.1. Effects of ATP Stimulation on Oxylipin Profiles in Astrocytes Adapted to High or Normal Glucose Concentrations

**Figure 1:**
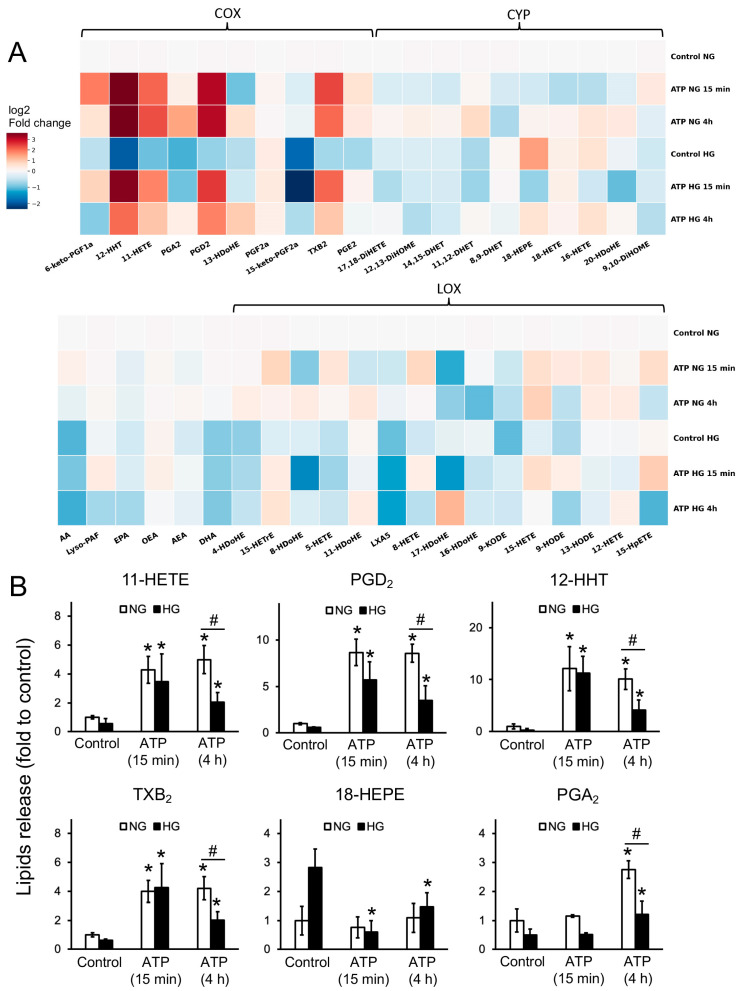
Effect of ATP stimulation on oxylipin release in astrocytes cultivated in HG and NG. Astrocytes were stimulated for 15 min or 4 h with ATP (100 μM). Concentrations of oxylipins in supernatants were measured using ultra-performance liquid chromatography–tandem mass spectrometry (UPLC-MS/MS). The heat map (**A**) shows relative amounts of each lipid mediator compared to the control. The X-axis shows the stimuli, while the Y-axis shows the fold change (log2 ratio scale) of each lipid mediator. Metabolites were divided based on the pathways involved in their synthesis: lipoxygenase (LOX), cyclooxygenase (COX), and cytochrome (CYP). (**B**) Relative concentration of 11-HETE, 18-HEPE, PGD2, 12-HHT, TXB2, and PGA2 metabolites. Values represent the mean ± SD from three independent experiments. * *p* < 0.05, compared with the unstimulated cells; # *p* < 0.05, compared with the NG-cultivated cells.

Oxylipins are an important part of the inflammatory response. We examined changes in the extracellular profiles of oxylipins during short-term (15 min) and medium-term (4 h) stimulation of astrocytes with ATP. Data for the 41 oxylipins and fatty acids analyzed are presented as a heat map (Figure 1A).

The detected compounds can be divided into the groups: (1) the cyclooxygenase (COX) pathway—prostaglandins (PG), thromboxane B2 (TXB2), 11-HETE (11-hydroxy-eicosatetraenoic acid), arachidonic acid (AA) derivatives, and 13-HDoHE (13-hydroxydocosahexaenoic acid, docosahexaenoic acid (DHA) derivative); (2) the cytochrome (CYP) pathway—dihydroxyeicosatetraenoic acid (17,18-DiHETE, eicosapentaenoic acid (EPA) derivative), dihydroxyoctadecenoic acids (9,10-/12,13-DiHOMEs, linoleic acid (LA) derivatives), dihydroxyeicosatrienoic acids (8,9-/11,12-DHETs, AA derivatives) (18-HEPE, EPA derivative), hydroxyeicosatetraenoic acids (16-/18-HETEs, AA derivatives), and hydroxydocosahexanoic acid (20-HDoHE, DHA derivative); (3) polyunsaturated fatty acids—AA, EPA, DHA, oleoylethanolamide (OEA), and anandamide (AEA); and (4) the lipoxygenase (LOX) pathway—hydroxydocosahexaenoic acids (4-/8-/11-/16-/17-HDoHEs, DHA derivatives), hydroxyeicosatrienoic acid (15-HETrE, dihomo-γ-linolenic acid derivative), 5-/8-/12-/15-HETE (AA derivatives), and hydroxyoctadecadienoic acids (9-/13-HODE, LA derivatives).

A 15 min ATP stimulation of astrocytes, grown in a medium with normal glucose, induced the release of COX derivatives such as PGD2, TXB2, 12-HHT, and 11-HETE (Figure 1B).

For cells adapted to a high-glucose medium (Figure 1B), there was also a significant increase in the COX derivatives PGD2, 12-HHT, and TXB2 and a significant decrease in the CYP derivative 18-HEPE (Figure 1B).

A four-hour ATP stimulation of astrocytes cultured in HG caused the release of the same oxylipins (Figure 1B), the levels of which were also altered by a 15 min stimulation: COX derivatives PGD2, 12-HHT, and TXB2 and LOX derivative 11-HETE (Figure 1B). The concentration of prostaglandin A2 (PGA2) was also significantly increased (Figure 1B).

Upon the stimulation of astrocytes cultured under HG conditions, an increase in the concentrations of PGD2, 12-HHT, TXB2, PGA2, and 11-HETE was observed (Figure 1B). Note that for each of these compounds, the increase was less than when cells were cultured in a medium with normal glucose levels. The amount of the CYP-derivative 18-HEPE decreased (Figure 1B), which is also consistent with the changes detected when analyzing the oxylipin profile during the 15 min ATP stimulation.

Thus, it can be concluded from the obtained data that both 4 h and 15 min stimulations of cells with ATP activate the COX pathway and increase the synthesis of AA-derivative metabolites but not the derivatives of other PUFAs. The synthesis of oxylipins upon the ATP stimulation of astrocytes is reduced when cells are adapted to high glucose.

### 3.2. Effect of Metformin on ATP-Stimulated Oxylipin Synthesis in Astrocytes Cultured at Different Glucose Concentrations

It was previously shown that metformin can have a significant effect on the response of astrocytes to the stimulation of TLR4 and TLR3 receptors; the effect depends on glucose concentration and is manifested in the synthesis of oxylipins [33,34]. Therefore, we evaluated the effect of metformin on the ATP-stimulated response of astrocytes. Cells were treated for 24 h with metformin and then stimulated with ATP for 15 min or 4 h, and 41 lipid compounds in the extracellular medium were measured. 

**Figure 2:**
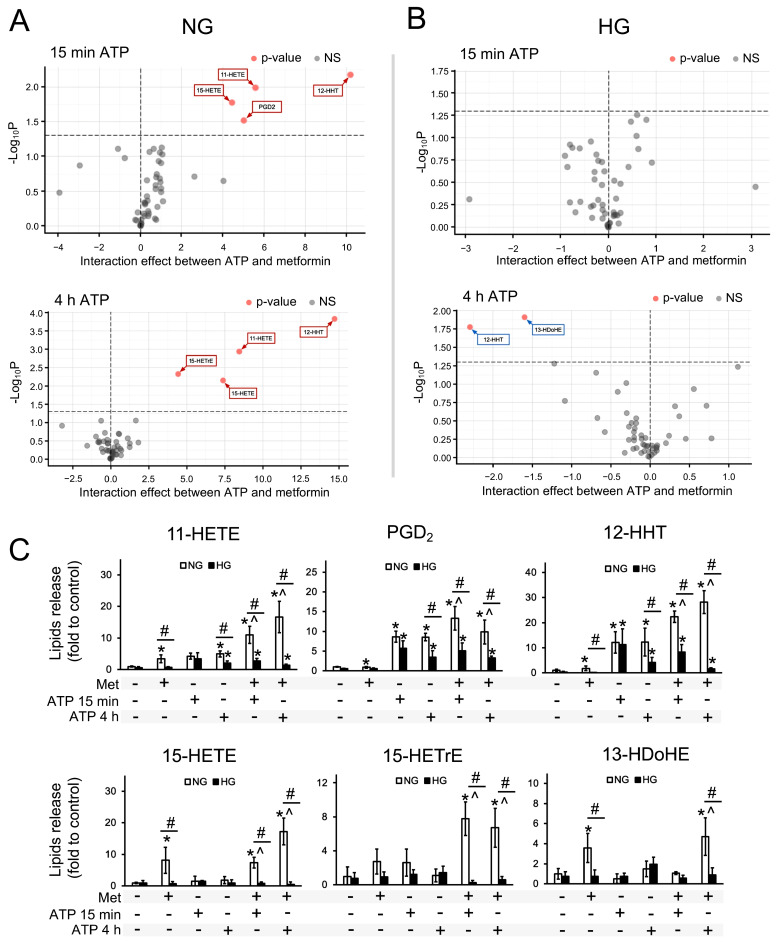
Effect of metformin (Met) on the secretion of oxylipins and PUFAs from astrocytes stimulated by ATP. Astrocytes were pretreated for 24 h with metformin (Met, 2.5 mM) and subsequently kept for 15 min or 4 h in ATP (100 μM). (**A,B**) Volcano plot highlighting compounds whose concentrations showed significant changes. Compounds with insignificant changes are marked in gray, while red dots stand for compounds that had a *p*-value (adjusted) less than 0.05. (**C**) Relative concentration of 11-HETE, PGD2, 12-HHT, 15-HETE, 15-HETrE, and 13-HDoHE metabolites. Values represent the mean ± SD from three independent experiments. * *p* < 0.05, compared with the unstimulated cells; # *p* < 0.05, compared with the NG-cultivated cells; ^ *p* < 0.05 for the hypothesis that both stimuli (ATP and Met) act independently for cells cultured in a medium with an appropriate glucose concentration (2-way ANOVA).

Since metformin affects oxylipin synthesis [33,34], to identify synergistic effects, we evaluated oxylipins for which the change in concentration upon combined ATP stimulation and metformin pretreatment was not explained by the independent effects of these factors. The results are presented as a volcano plot (Figure 2A,B) showing oxylipins for which an interaction between factors such as ATP stimulation and metformin pretreatment was observed for astrocytes cultured in a medium with normal (Figure 2A) and high (Figure 2B) glucose concentrations. The Y-axis indicates log10 *p*-values at the significance level of the hypothesis that ATP and metformin have independent effects. The X-axis indicates a fold change in the interaction effect of the short-term stimulation with ATP and pretreatment with metformin, which was calculated as the difference between the mean concentration during treatment with both stimuli and the concentration during treatment with each stimulus separately, summed with the concentration of the corresponding lipid mediator in control cells. The positive values of this expression mean that the interaction between the stimuli resulted in a higher lipid mediator level than would have been the case if the stimuli had been treated independently, and negative values mean that one is lower. Red dots stand for compounds that had a *p*-value (adjusted) < 0.05. Compounds that changed insignificantly are indicated in gray.

For astrocytes cultured in a normal glucose medium, synergistic effects caused by the 15 min stimulation with ATP and metformin were observed for a range of oxylipins (Figure 2A,C), such as the cyclooxygenase derivatives 11-HETE, 12-HHT, and PGD2 and the lipoxygenase derivative 15-HETE. For these lipid mediators, the concentrations when co-stimulated by ATP and metformin were significantly higher than the sum of the effects of these stimuli separately. 

For cells cultured in a medium with normal glucose (Figure 2A,C), a synergistic effect caused by metformin and ATP (4 h) on the COX derivatives 12-HHT and 11-HETE and the LOX derivative 15-HETE was observed, as well as during short-term stimulation. At the same time, no interaction effects were observed for PGD2, but a synergistic effect was observed for the lipoxygenase pathway derivative 15-HETrE. 

It is noteworthy that during the adaptation of astrocytes to hyperglycemic conditions, no interaction effects between ATP at 15 min and metformin were observed for any of the analyzed oxylipins (Figure 2B,C). More significant changes were observed for different oxylipins at four hours of ATP stimulation than were obtained with short-term stimulation. No synergistic effects were observed for any of the lipid mediators studied. The concentration of 12-HHT decreased under the combined effect of ATP and metformin, while the opposite (synergistic) effect for this oxylipin was observed for cells cultured in a medium with a normal glucose concentration.

### 3.3. Alterations to the Levels of Inflammatory Markers in ATP-Stimulated Astrocytes Cultured at High Glucose Concentrations

**Figure 3:**
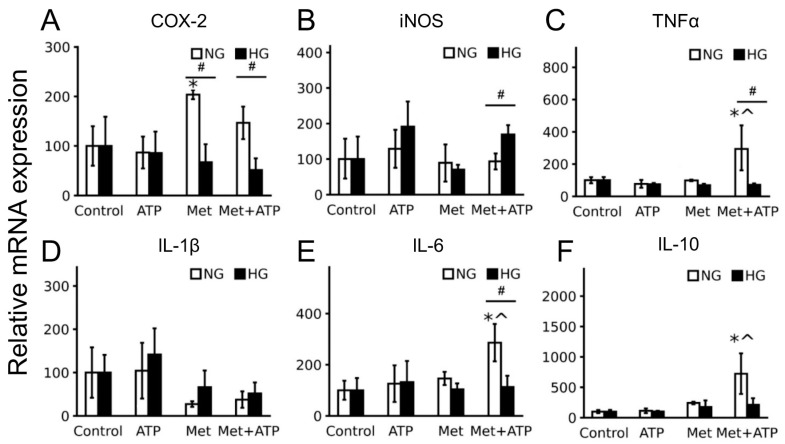
High glucose does not influence astrocyte sensitivity for ATP but reveals various effects of metformin. Astrocytes were pretreated for 24 h with metformin (Met, 2.5 mM) and subsequently kept for 4 h in ATP (100 μM). (**A**–**F**) mRNA levels of inflammatory markers TNFα, IL-1β, iNOS, IL-10, COX-2, and IL-6 were determined by quantitative real-time PCR (qPCR). The results are expressed as a % (expression in the control = 100%). The values represent a mean ± SD from three independent experiments. * *p* < 0.05, compared with the unstimulated cells; # *p* < 0.05, compared with the NG-cultivated cells; ^ *p* < 0.05, compared with the ATP-stimulated cells.

To characterize the effect of ATP on astrocyte inflammatory responses, we assessed the expression level of pro-inflammatory interleukins IL-6 and IL-1β, cytokine tumor necrosis factor-alpha (TNFα), pro-inflammatory enzymes iNOS (inducible NO synthase) and COX-2 (cyclooxygenase 2), and anti-inflammatory interleukin 10 (IL-10) (Figure 3). The stimulation of astrocytes with ATP did not induce an increase in the expression of inflammatory markers for normal or high glucose. The pretreatment of astrocytes with metformin increased COX-2 expression for cells cultured in normal glucose medium (Figure 3A). The co-stimulation of astrocytes with ATP and metformin increased the expressions of TNFα (Figure 3C), IL-6 (Figure 3E), and IL-10 (Figure 3F) for cells cultured in a medium with normal glucose. No effects of ATP and metformin were observed for HG-cultivated cells.

### 3.4. Intracellular Mechanisms Involved in the ATP-Stimulated Response of Astrocytes During Adaptation to High Glucose and Pretreatment with Metformin

**Figure 4:**
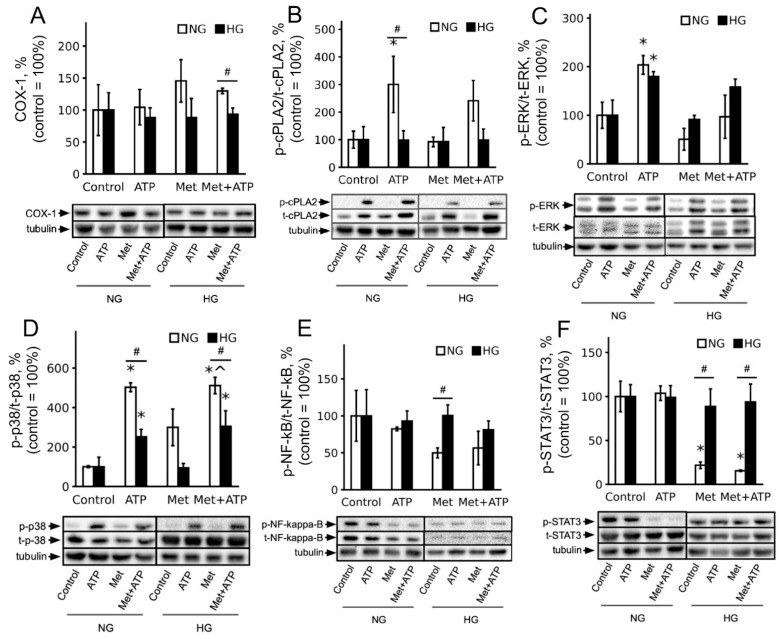
Comparison of COX-1, cPLA2, ERK1/2, p38 MAPK, NF-κB, and STAT3 activity in ATP-stimulated astrocytes treated with metformin. Astrocytes were pretreated for 24 h with metformin (Met, 2.5 mM) and subsequently kept for 15 min in ATP (100 μM). (**A**–**F**) COX-1, p-cPLA2, cPLA2, p38, p-p38, b p-ERK1/2, ERK1/2, p-STAT3, STAT3, p-NF-κB, and NF-κB protein levels were evaluated by Western blotting (Supplementary Materials) and normalized to the loading control, β-tubulin. The example is representative of three independent experiments. Results are expressed as fold changes, relative to untreated cells. Values represent mean ± SD from three independent experiments. * *p* < 0.05, compared with the control cells; # *p* < 0.05, compared with the NG-cultivated cells; ^ *p* < 0.05, compared with the ATP-stimulated cells.

To elucidate the intracellular molecular mechanisms underlying the variations in the astrocyte’s responses, the activity of intracellular markers was evaluated under stimulation with 100 μM ATP for 15 min (Figure 4). 

Cyclooxygenase 1 (COX-1, Figure 4A) and the relative amounts of the active form of cytosolic phospholipase A2 (cPLA2, Figure 4B) were analyzed to determine the mechanisms involved in the changes in oxylipin profiles. COX-1 levels were unchanged by ATP stimulation for both glucose concentrations. The active form of cytosolic phospholipase A2 increased in response to ATP in astrocytes cultured in an NG medium (Figure 4B). No effect of ATP was observed on cells with high glucose (Figure 4B). Metformin did not affect COX-1 or cPLA2 (Figure 4A,B).

ATP stimulates the phosphorylation of extracellular-signal-regulated kinase ERK1/2 (Figure 4C) and p38 mitogen-activated protein kinase (MAPK) (Figure 4D). ERK1/2 phosphorylation was independent of the glucose level in the medium in which the cells were cultured. The pretreatment of astrocytes with metformin decreased ERK1/2 phosphorylation in both native and ATP-stimulated cells in normal glucose (Figure 4C). The pretreatment of cells with metformin increased p38 phosphorylation in NG. The activation of p38 MAPK in response to ATP was reduced in high glucose (Figure 4D). Metformin did not affect the ATP-stimulated phosphorylation of p38 (Figure 4D).

The levels of the phosphorylated form of the transcription factor NF-κB (Figure 4E) and the signal transducer and activator of transcription-3 (STAT3) (Figure 4F) were not altered by ATP. The pretreatment of cells with metformin significantly reduced STAT3 but not NF-κB levels, and this effect was only evident for cells cultured in a medium with normal glucose (Figure 4F). The inhibitory effect of metformin was independent of the presence of ATP (Figure 4E,F).

## 4. Discussion

Although extracellular ATP has been extensively discussed as a participant in the inflammatory process in astrocytes [1,6], our data indicate that its role is more complex. ATP does not induce changes in the expression of pro-inflammatory markers (COX-2, iNOS, TNFα, IL-1β, IL-6, and IL-10) but does affect the synthesis of oxylipins (PGD2, TxB2, 12-HHT, 11-HETE, PGA2, and 18-HEPE). Previously, there have been data on the effect of ATP on the synthesis of individual prostaglandins; for example, an increase in PGD2 was observed in [36], TXB2 in [37], and PGE2 in [38]. In these studies, the determination of prostaglandins was performed in a serum-free medium, which affects the profile of oxylipins synthesized by astrocytes, as we have shown previously [21]. Presently, it is difficult to assess whether the change in oxylipin profiles observed with ATP action is a shift toward increased inflammation or its resolution. We can still only discuss our knowledge of the action of individual substances. TXB2 (or its unstable precursor, TxA2) and 11-HETE are mainly considered pro-inflammatory substances [39]. Prostaglandin D2 (PGD2), the most abundant prostaglandin in the brain, may exert pro-inflammatory or anti-inflammatory effects by acting via different receptors or transformations for cyclopentenone prostaglandins [40]. Cyclopentenone PGA2, a derivative of PGE2, is considered an anti-inflammatory compound [41]. 12-HHT [42] and 18-HEPE [43] both exhibit anti-inflammatory properties. Although many individual oxylipins are released at low concentrations, it was previously hypothesized that their effects may add up, and low concentrations of oxylipins can be summarized [44]. Taking this into account, it can be assumed that the ATP-stimulated oxylipin spectrum shifts toward anti-inflammatory activity. The appearance of PGA2 upon prolonged exposure to ATP also confirms this assumption; i.e., the shift of ATP-induced oxylipin synthesis toward an anti-inflammatory profile is further enhanced.

ATP is a gliotransmitter, and its sustained increase has been observed in various pathological processes in the CNS [1,5,7]; therefore, it has been suggested as a participant in the pro-inflammatory part of the inflammatory response, although our data suggest that ATP is a participant in resolution processes. Our results with inflammatory markers are also in line with oxylipin data and cast doubt on the assertion of ATP as a pro-inflammatory agent. Indeed, we see no effect from ATP on the expression of COX-2, a well-known inducible key enzyme of arachidonic acid conversion into prostaglandins, 11-HHT, and other oxylipins [17]. Other pro-inflammatory markers, i.e., iNOS, TNFα, IL-1β, IL-6, and IL-10, were not stimulated by ATP even after 4 h of treatment. The lack of an effect on marker expressions correlates well with the lack of effect from ATP on the activation of the pro-inflammatory gene transcription factor NF-κB. Our data are in line with others; it has been shown that ATP does not induce the release of L-1β [45], TNFα [46], or inducible nitric oxide synthase iNOS [47]. 

It has been shown that the prolonged culturing of astrocytes in high glucose increases ATP release [15]. Data obtained in endothelial cells suggest that high glucose levels may directly affect the sensitivity of P2X7 and P2X4 receptors to ATP [16]. One would expect that ATP action would be reduced in HG-adapted astrocytes. Indeed, we have observed decreased activity for cPLA2 and p38 for HG. Also, oxylipin synthesis activity was reduced by prolonged treatment (4 h) with ATP for HG. However, short stimulation (15 min) with ATP did not reveal any difference in the effects of ATP on oxylipin synthesis in NG or HG. This does not support the hypothesis of the decreased sensitivity of purinergic receptors to ATP in astrocytes during adaptation to HG. Additional investigations are required for the characterization of possible changes in the activity of purinergic receptors during the adaptation of astrocytes to high glucose. It is notable that the sensitivity of cells to the action of ATP changes during adaptation to HG, but these appear to be quantitative rather than qualitative changes.

Metformin has attracted attention due to its anti-inflammatory activity and therapeutic potential in clinical fields other than type 2 diabetes [28]. We have previously shown that the efficacy of metformin in astrocytes is dependent on glucose concentrations [33]. Metformin notably decreased both basal respiration and the maximum respiration rate after the uncoupler, as well as oligomycin-sensitive respiration, regardless of the glucose concentration in the medium. However, it enhanced the free respiratory capacity of astrocytes only in HG [33]. Pretreating astrocytes with metformin raised the level of basal glycolysis but had no impact on glycolytic capacity, and both were unaffected by the glucose concentration in the medium [33].

We previously evaluated the potential of metformin as an anti-inflammatory agent in the action of toll-like receptor (TLR) stimuli, that is, TLR4 [33] and TLR3 [34]. Cell cultivation was carried out under the same experimental conditions as in the present study. Metformin decreased the release of IL-1β and IL-6, and the activity of the transcription factor STAT3 and ERK MAPK was triggered by lipopolysaccharide (LPS) independently of glucose concentration [33]. Activating TLR3 reveals the dependence of metformin’s effects on glucose concentrations. In this study, metformin reduced the Poly I:C (PIC)-stimulated expression of IL-1β independently of glucose concentration but decreased TNFα expression and IL-6 release only in NG. Metformin significantly activated p38 MAPK and reduced STAT3 activity in PIC-stimulated cells independently of glucose concentration [34]. In the present study, we have shown that metformin affects ERK, STAT3, and NF-κB activity independent of the presence of ATP. The effects of metformin were evident in NG but not in HG-cultivated cells. A comparison of the effects of metformin on TLR-stimulated cells and ATP-stimulated cells further indicates that ATP cannot be attributed to a pure pro-inflammatory stimulus on astrocytes. 

It is also interesting to compare the effects of metformin on oxylipin synthesis for these stimuli. Metformin reduced the LPS-stimulated release of COX-derived oxylipins and anandamide independent of the glucose concentration. Different effects of glucose adaptation were observed when oxylipin release was analyzed during PIC stimulation. Metformin reduced the PIC-stimulated synthesis of COX-derived oxylipins in the HG (11-HETE, 6-keto-PGF1α, PGD2, TXB2, 13-HDoHE) and increased/did not affect it in the NG model (11-HETE, 6-keto-PGF1α, TXB2, 13-HDoHE). Using ATP as a stimulus, we found a potentiating effect for metformin (increased release of 11-HETE, PGD2, 12-HHT, 15-HETE, 13-HDoHE, 15-HETrE) that was predominantly evident in NG-cultivated cells. The results suggest that metformin may be considered not only an anti-inflammatory drug but also a drug that promotes the resolution of inflammation.

## 5. Conclusions

Our data provide evidence that ATP induces the release of anti-inflammatory oxylipins independent of high or normal glucose concentrations; metformin enhances these effects in normal glucose. Compared to inflammatory stimuli such as TLR3 and TLR4 agonists, extracellular ATP does not exhibit the properties of a proinflammatory stimulus since it does not activate cytokines and other classical markers of the inflammatory response. ATP remains an active regulator of astrocyte responses, altering the activity of intracellular proteins when cultured in normal but not high glucose. The functions of increasing the concentration of ATP in neuroinflammation should be considered from the perspective of its role as a participant in the resolution processes of neuroinflammation. These results may be advised in the development of anti-inflammatory therapeutic approaches aimed at modulating astrocyte function in various pathologies.

## Figures and Tables

**Table 1:** DNA sequences of the primers used for RT-PCR.

Name	Forward (5’-3’)	Reverse (5’-3’)	NM Accession Number	Amplicon
*TNFα*	CAAGGAGGAGAAGTTCCCAA	TGATCTGAGTGTGAGGGTCTG	012675	70
*IL-10*	CCCAGAAATCAAGGAGCATTTG	TCATTCTTCACCTGCTCCAC	012854	129
*COX-2*	TGTACAAGCAGTGGCAAAGG	TAGCATCTGGACGAGGCTTT	017232	221
*IL-1β*	CACCTCTCAAGCAGAGCACAG	GGGTTCCATGGTGAAGTCAAC	031512	79
*IL-6*	CTGGTCTTCTGGAGTTCCGT	TGGTCTTGGTCCTTAGCCAC	012589	225
*iNOS*	CCACAATAGTACAATACTACTTGG	ACGAGGTGTTCAGCGTGCTCCACG	012611	394
*β-actin*	AGATGACCCAGATCATGTTTGAG	GGCATACAGGGACAACACAG	031144	80

## Data Availability

All data and materials used are available from the corresponding author upon reasonable request. The data are not publicly available due to privacy.

## Supplementary Materials

The following supporting information can be downloaded at: https://www.mdpi.com/article/10.3390/brainsci15030293/s1, Figure S1. Comparison of COX-1, cPLA2, ERK1/2 and p38 MAPK, NF-κB and STAT3 activity in the ATP-stimulated astrocytes, treated with metformin. Astrocytes were pretreated for 24 h with metformin (Met, 2.5 mM) and subsequently kept for 15 min with ATP (100 μM). COX-1, p-cPLA2, cPLA2, p38, pp38, p-ERK1/2, ERK1/2, p-STAT3, STAT3, p-NF-κB, and NF-κB protein levels were evaluated by western blotting.

## Abbreviations

The following abbreviations are used in this manuscript:

AA	arachidonic acid
COX	cyclooxygenase
DHA	docosahexaenoic acid
EPA	eicosapentaenoic acid
HG	high glucose concentration
NG	normal glucose concentration
LPS	lipopolysaccharide
IL	interleukin
PG	prostaglandin
PUFA	polyunsaturated fatty acid
LOX	lipoxygenase
FBS	fetal bovine serum
HETE	hydroxyeicosatetraenoic acid
HDoHE	hydroxydocosahexaenoic acid
HHT	hydroxyheptadecatrenoic acid
