# Supplementary material for: ATP Alters the Oxylipin Profiles in Astrocytes: Modulation by High Glucose and Metformin
Source: Brain Sci. 2025 Mar 11;15(3):293. doi: 10.3390/brainsci15030293 (PMC11940397; doi:10.3390/brainsci15030293)
Supplement: Supplementary file 1 [file brainsci-15-00293-s001.zip › Figure_S1.pdf]

Normal glucose (5 mM)

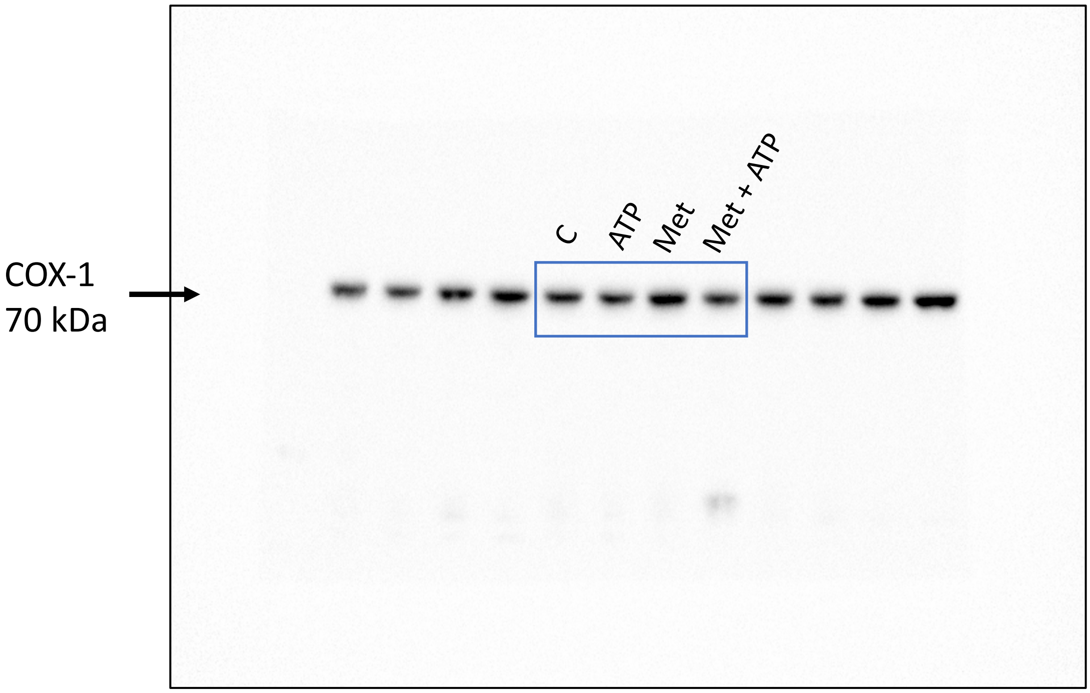

Cox1 Antibody #4841 (Cell Signaling Technology, Danvers, MA, USA)

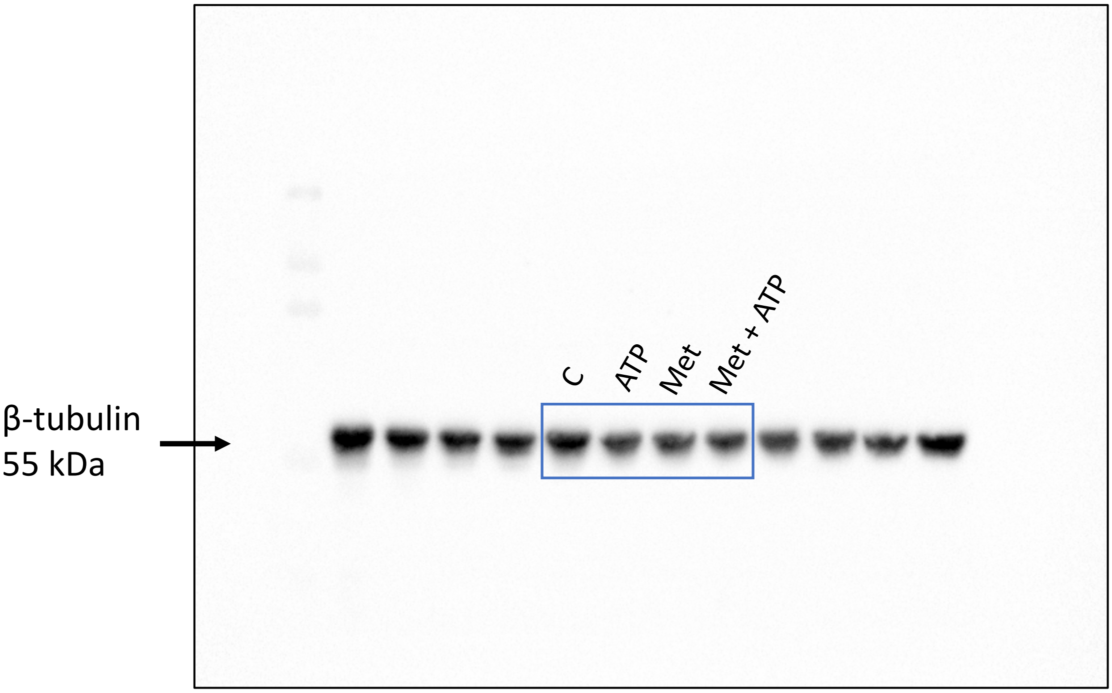

β-tubulin (D3U1W) #86298 (Cell Signaling Technology, Danvers, MA, USA)

High glucose (22.5 mM)

COX-1
70 kDa

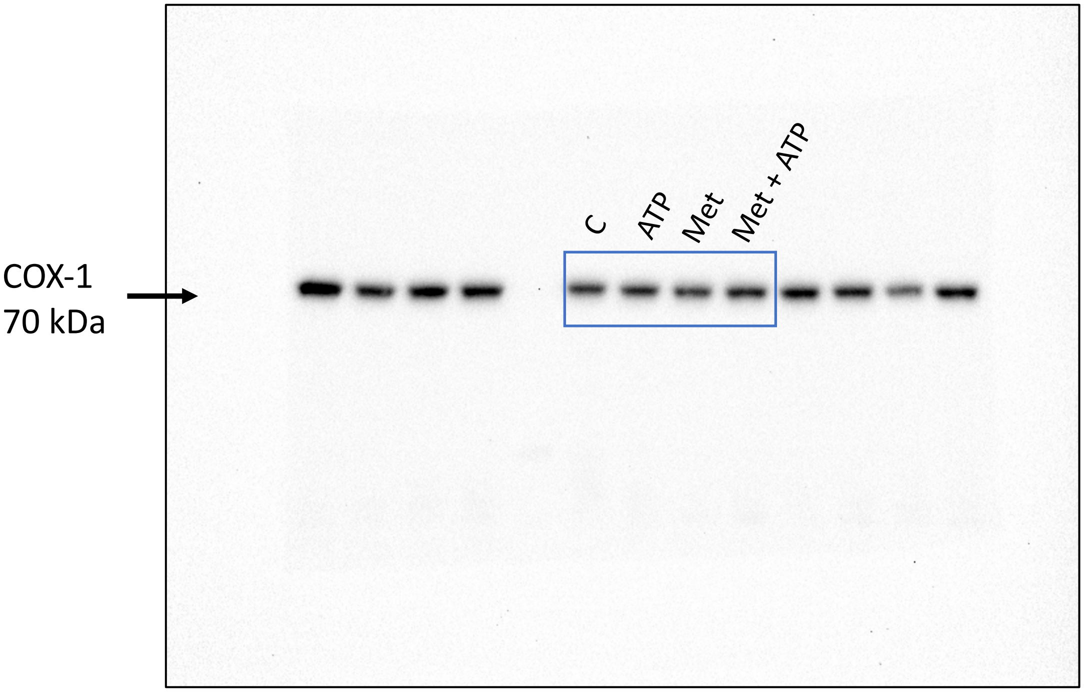

Cox1 Antibody #4841 (Cell Signaling Technology, Danvers, MA, USA)

β-tubulin
55 kDa

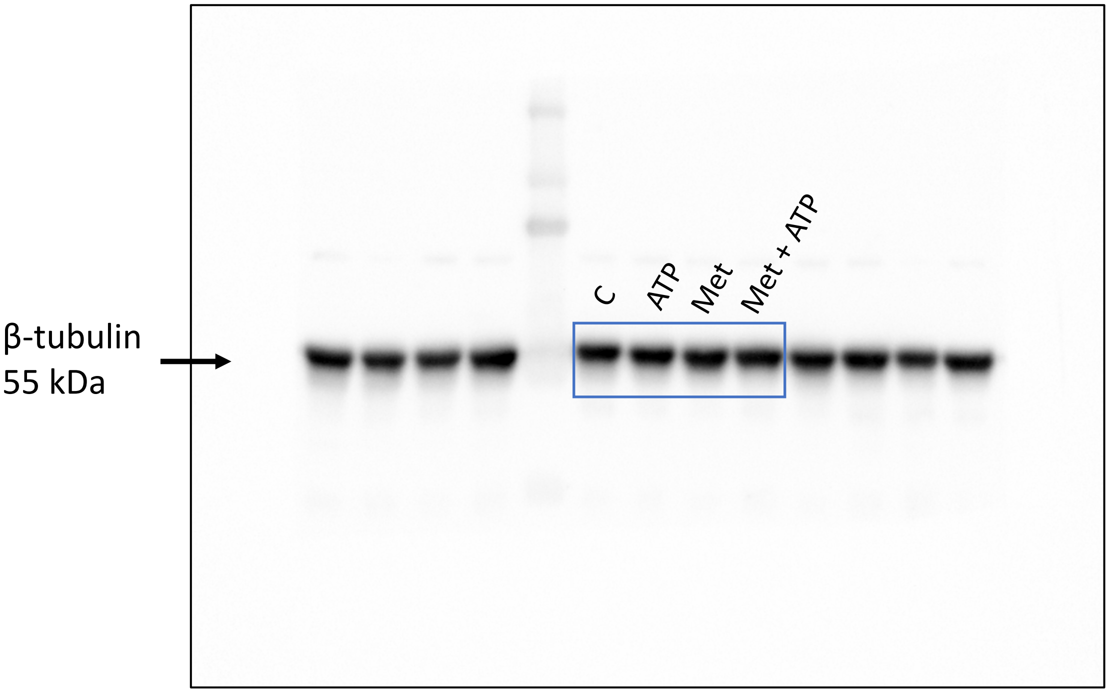

β-tubulin (D3U1W) #86298 (Cell Signaling Technology, Danvers, MA, USA)

Normal glucose (5 mM)

p-cPLA2
95 kDa

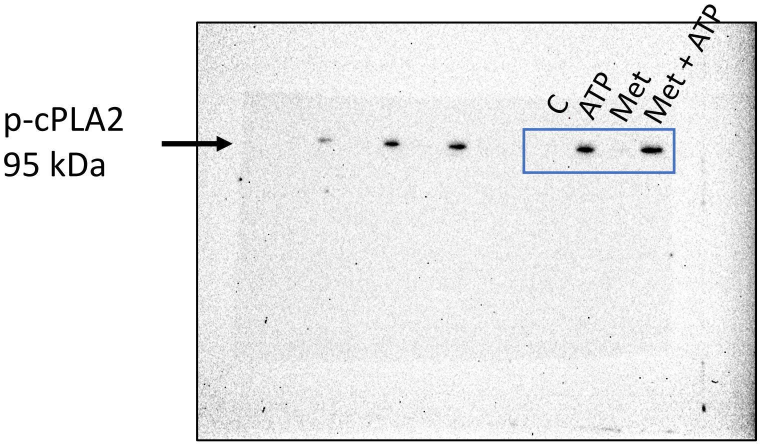

Phospho-cPLA2 (Ser505) Antibody #2831 (Cell Signaling Technology, Danvers, MA, USA)

cPLA2
95 kDa

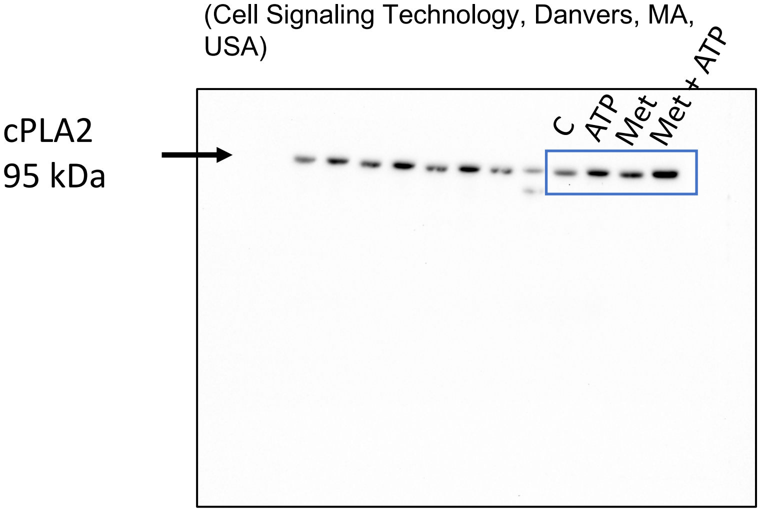

cPLA2 Antibody #2832 (Cell Signaling Technology, Danvers, MA, USA)

β-tubulin
55 kDa

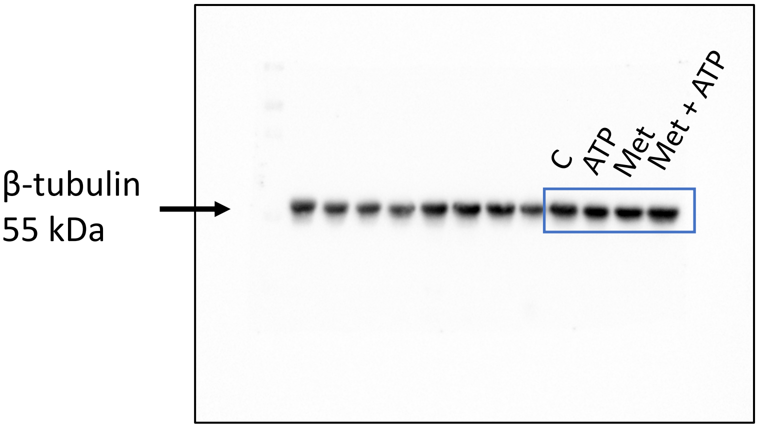

β-tubulin (D3U1W) #86298 (Cell Signaling Technology, Danvers, MA, USA)

High glucose (22.5 mM)

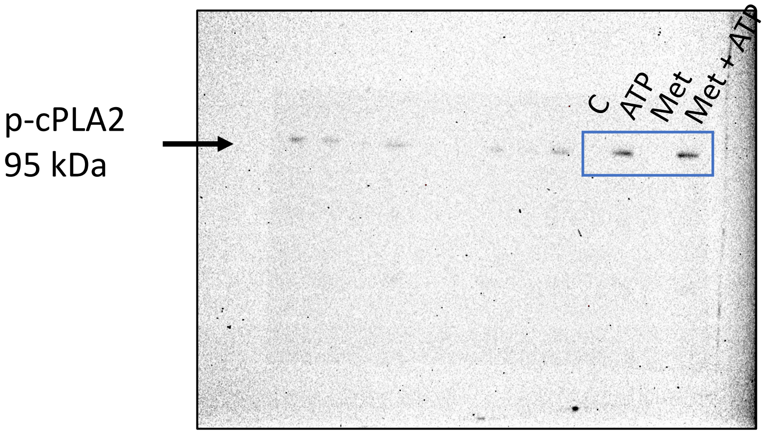

Phospho-cPLA2 (Ser505) Antibody #2831 (Cell Signaling Technology, Danvers, MA, USA)

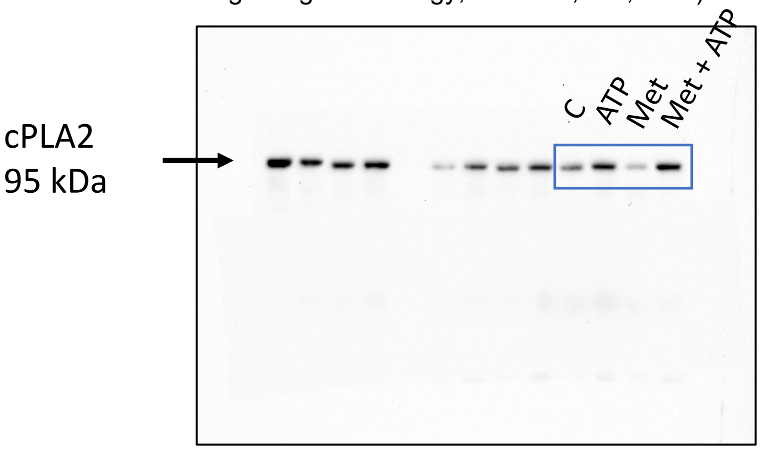

cPLA2 Antibody #2832 (Cell Signaling Technology, Danvers, MA, USA)

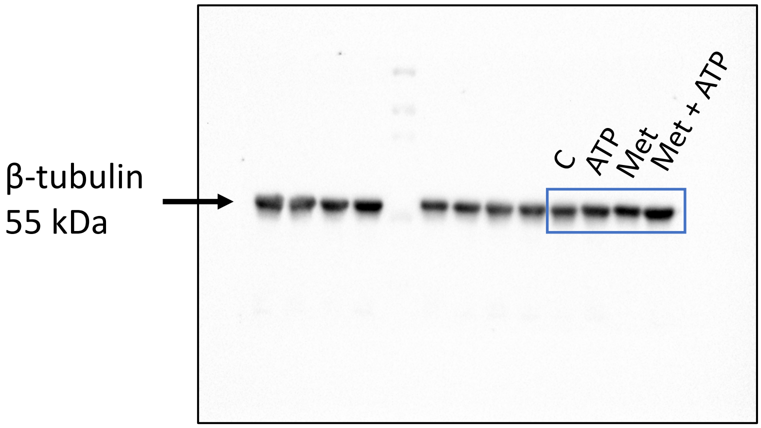

β-tubulin (D3U1W) #86298 (Cell Signaling Technology, Danvers, MA, USA)

Normal glucose (5 mM)

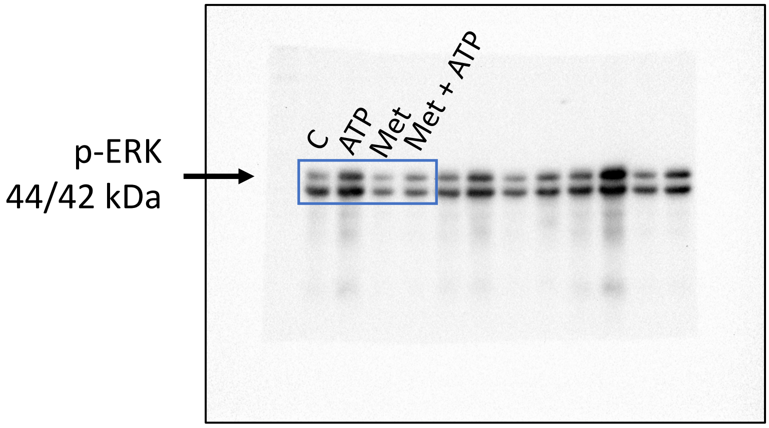

Phospho-p44/42 MAPK (Erk1/2) (Thr202/Tyr204) #4370 (Cell Signaling Technology, Danvers, MA, USA)

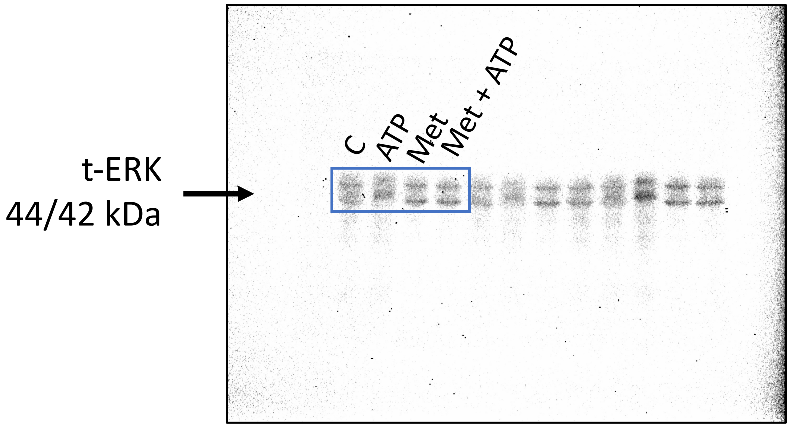

p44/42 MAPK (Erk1/2) Antibody #9102 (Cell Signaling Technology, Danvers, MA, USA)

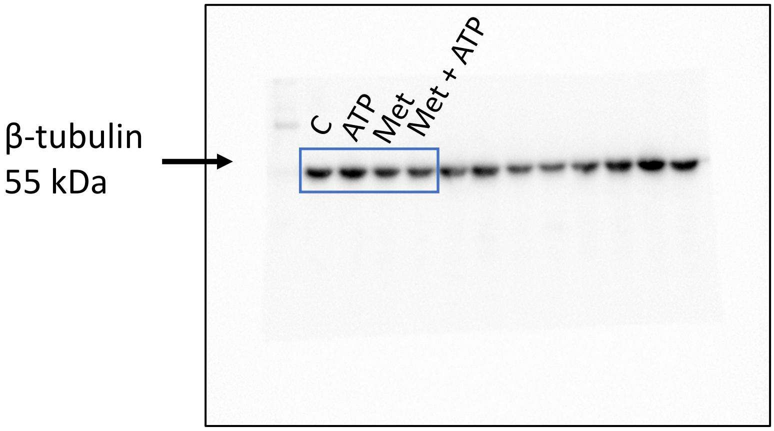

β-tubulin (D3U1W) #86298 (Cell Signaling Technology, Danvers, MA, USA)

High glucose (22.5 mM)

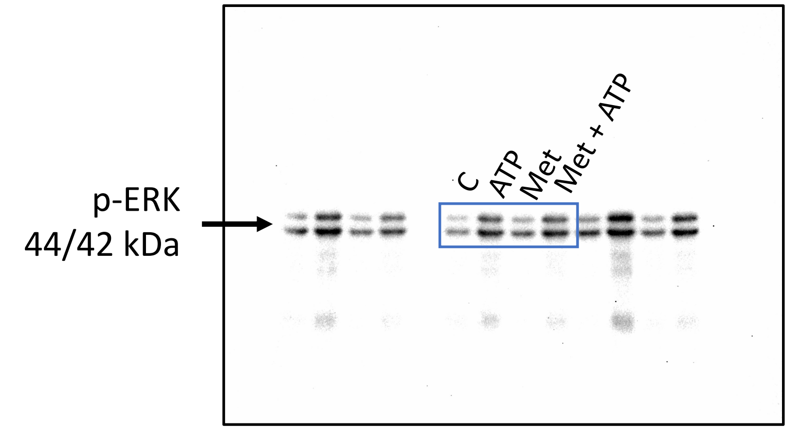

Phospho-p44/42 MAPK (Erk1/2) (Thr202/Tyr204) #4370 (Cell Signaling Technology, Danvers, MA, USA)

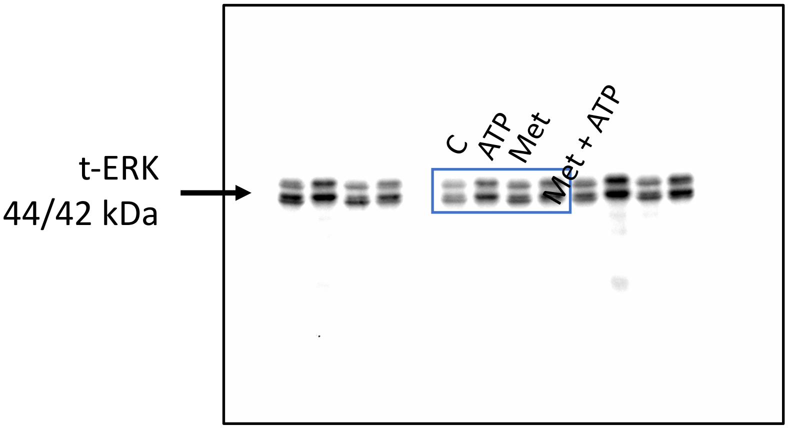

p44/42 MAPK (Erk1/2) Antibody #9102 (Cell Signaling Technology, Danvers, MA, USA)

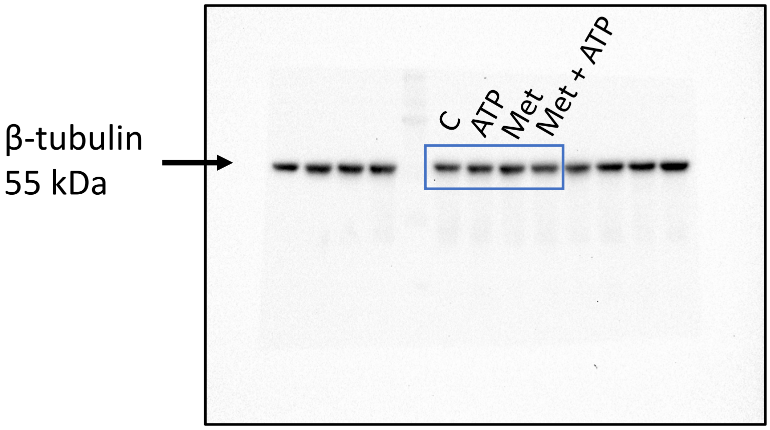

β-tubulin (D3U1W) #86298 (Cell Signaling Technology, Danvers, MA, USA)

Normal glucose (5 mM)

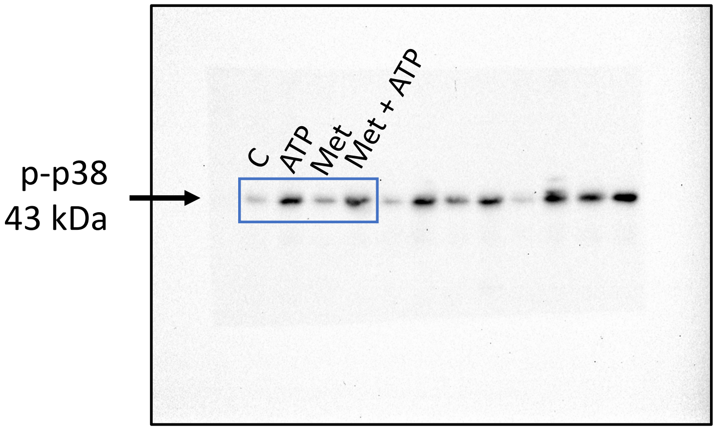

Phospho-p38 MAPK (Thr180/Tyr182) Antibody #9211
(Cell Signaling Technology, Danvers, MA, USA)

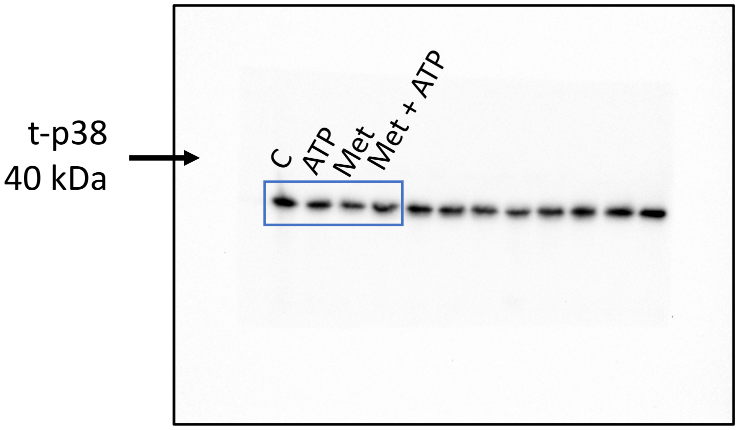

p38 MAPK Antibody #9212 (Cell Signaling
Technology, Danvers, MA, USA)

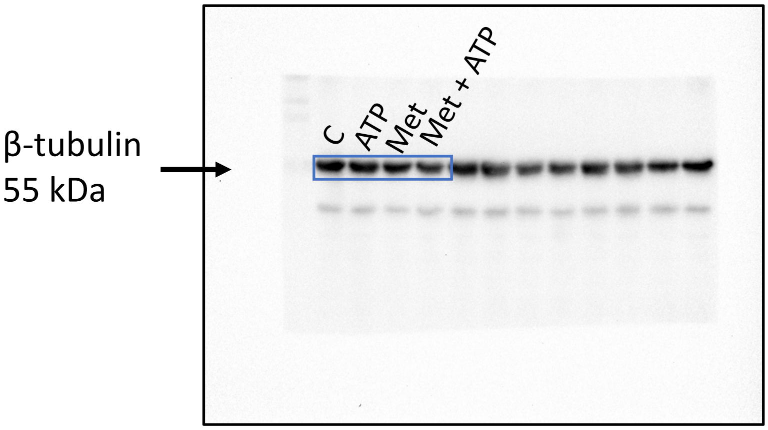

β-tubulin (D3U1W) #86298 (Cell Signaling
Technology, Danvers, MA, USA)

High glucose (22.5 mM)

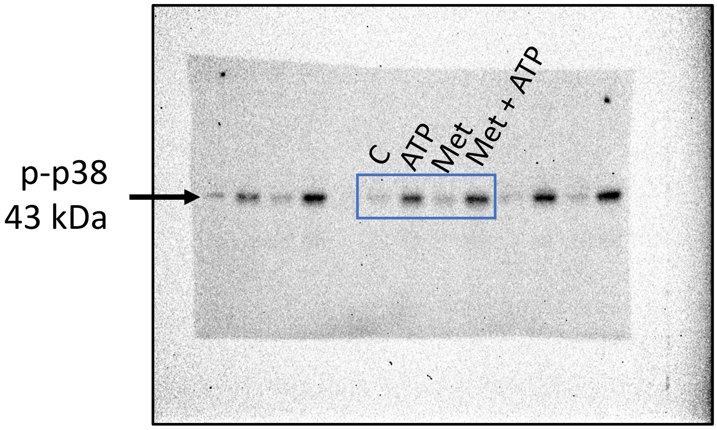

Phospho-p38 MAPK (Thr180/Tyr182) Antibody #9211 (Cell Signaling Technology, Danvers, MA, USA)

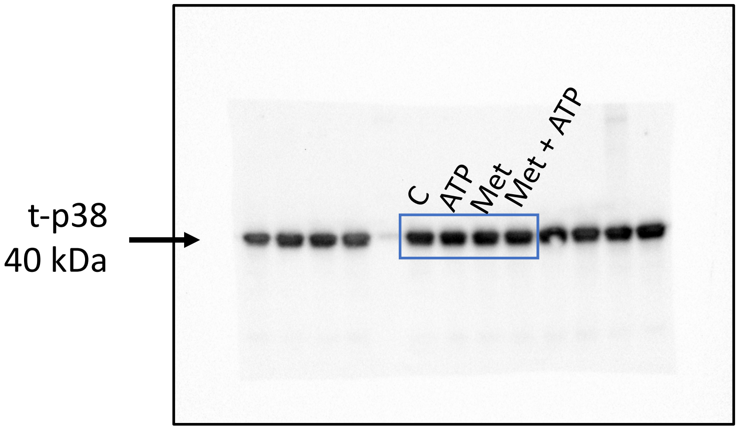

p38 MAPK Antibody #9212 (Cell Signaling Technology, Danvers, MA, USA)

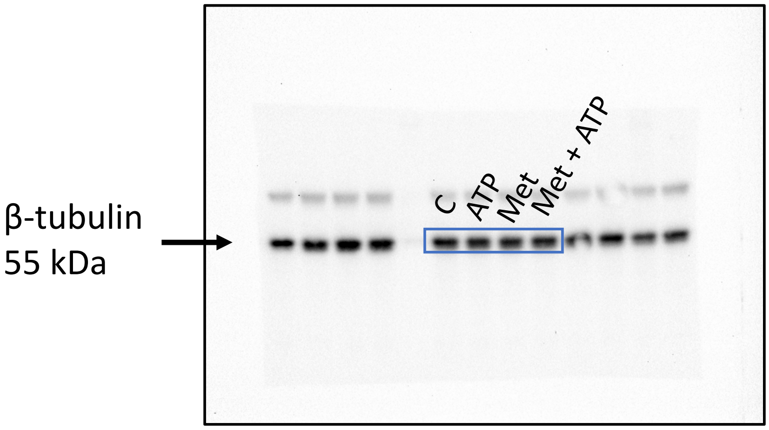

β-tubulin (D3U1W) #86298 (Cell Signaling Technology, Danvers, MA, USA)

Normal glucose (5 mM)

p-NF-kB
65 kDa

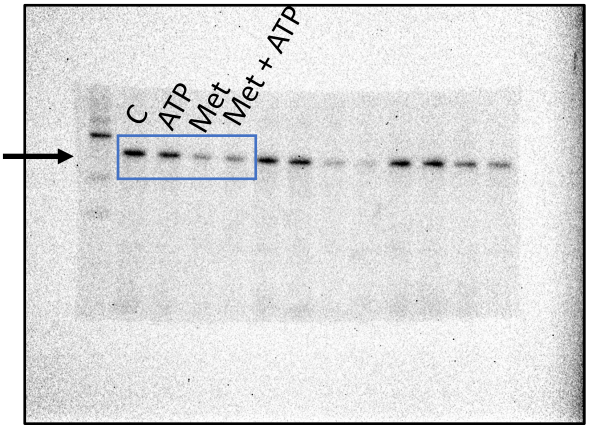

Phospho-NF-κB p65 (Ser536) (93H1) Rabbit mAb #3033 (Cell Signaling Technology, Danvers, MA, USA)

t-NF-kB
65 kDa

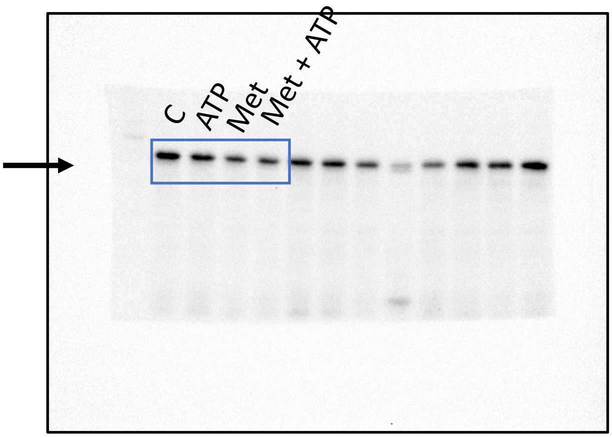

NF-κB p65 (D14E12) XP® Rabbit mAb #8242 (Cell Signaling Technology, Danvers, MA, USA)

β-tubulin
55 kDa

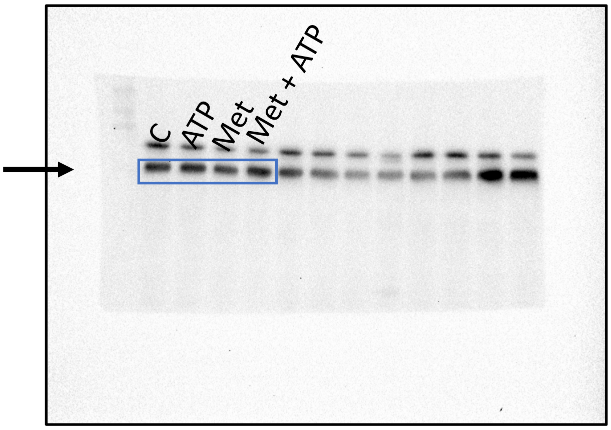

β-tubulin (D3U1W) #86298 (Cell Signaling Technology, Danvers, MA, USA)

High glucose (22.5 mM)

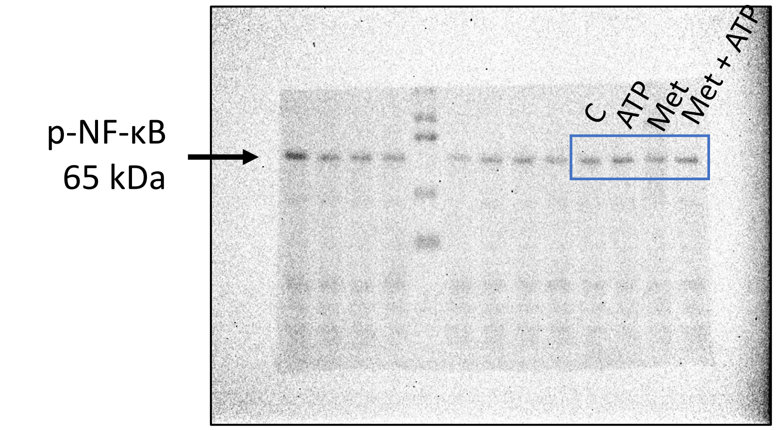

Phospho-NF-κB p65 (Ser536) (93H1) Rabbit mAb #3033 (Cell Signaling Technology, Danvers, MA, USA)

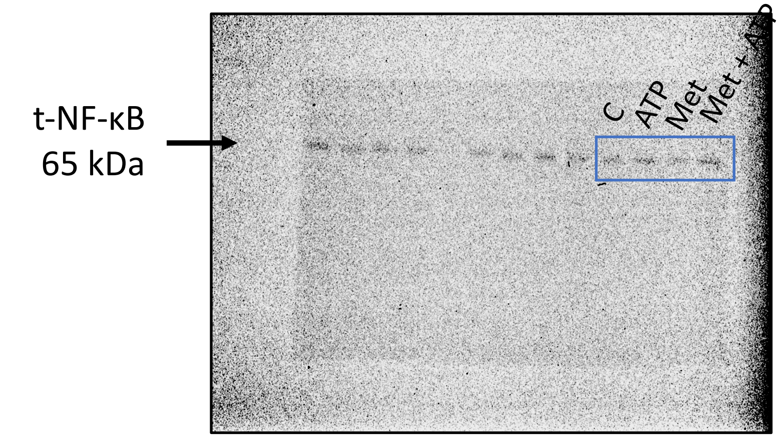

NF-κB p65 (D14E12) XP® Rabbit mAb #8242 (Cell Signaling Technology, Danvers, MA, USA)

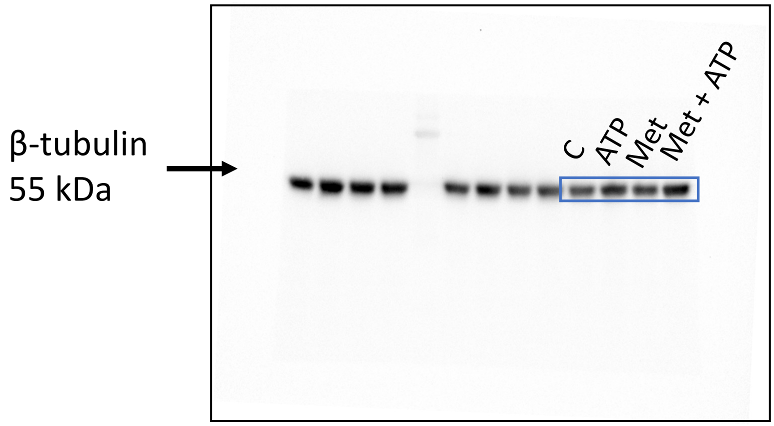

β-tubulin (D3U1W) #86298 (Cell Signaling Technology, Danvers, MA, USA)

Normal glucose (5 mM)

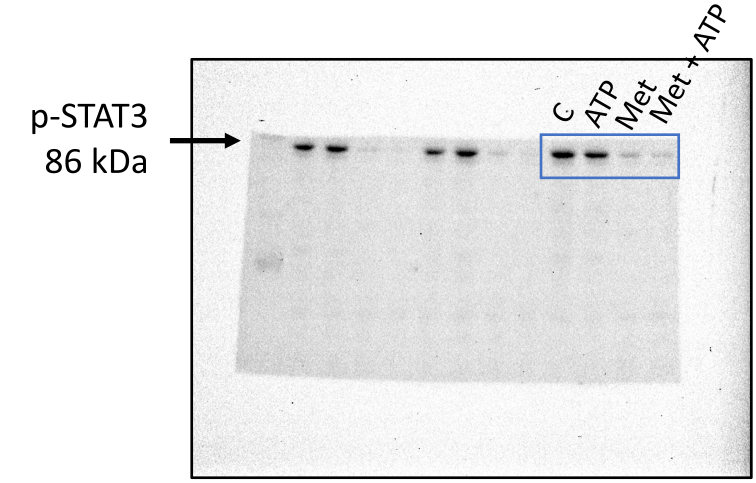

Phospho-Stat3 (Tyr705) (D3A7) XP® Rabbit mAb #9145

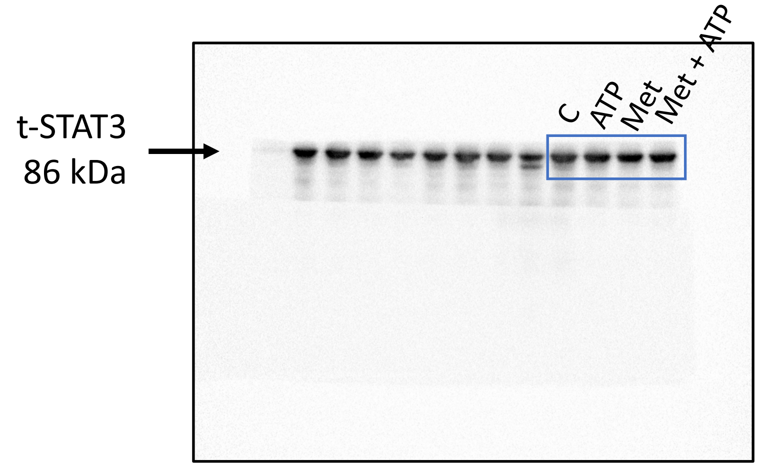

Stat3 (D3Z2G) Rabbit mAb #12640 (Cell Signaling Technology, Danvers, MA, USA)

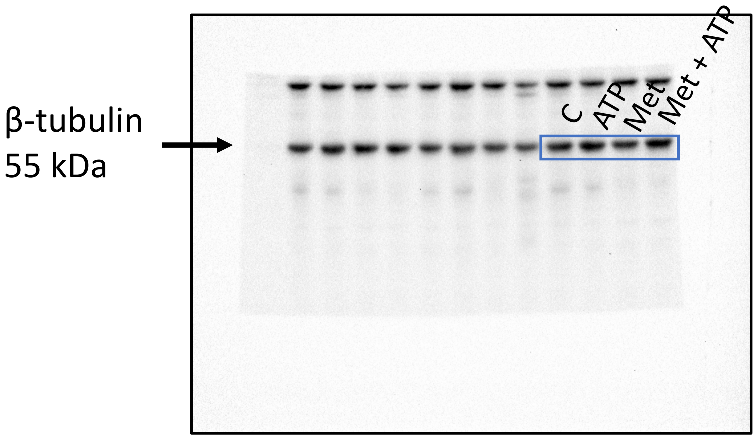

β-tubulin (D3U1W) #86298 (Cell Signaling Technology, Danvers, MA, USA)

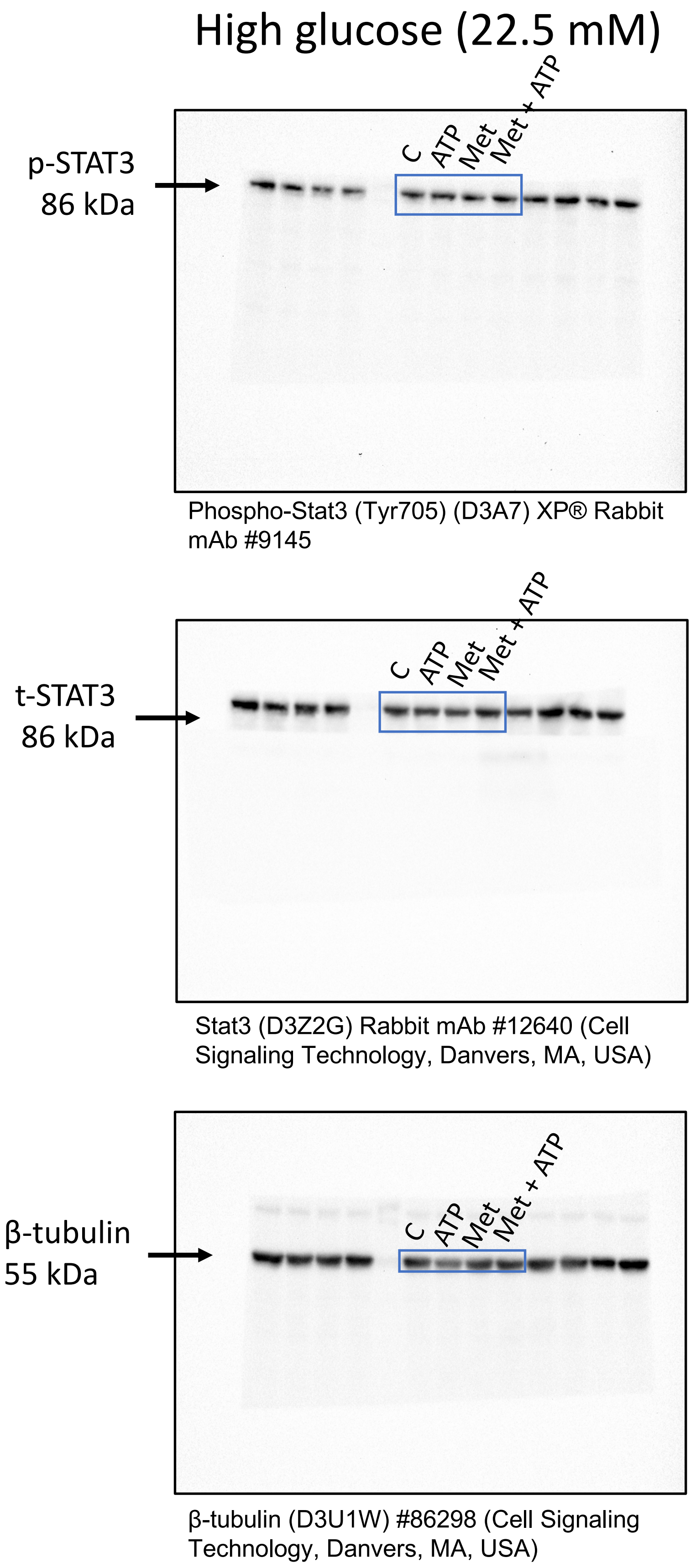

Figure S1. Comparison of COX-1, cPLA2, ERK1/2 and p38 MAPK, NF-κB and STAT3 activity in the ATP-stimulated astrocytes, treated with metformin. Astrocytes were pretreated for 24 h with metformin (Met, 2.5 mM) and subsequently kept for 15 min with ATP (100 μM). COX-1, p-cPLA2, cPLA2, p38, p-p38, p-ERK1/2, ERK1/2, p-STAT3, STAT3, p-NF-κB, and NF-κB protein levels were evaluated by western blotting.
